# New Approaches to Manage Infections in Transplant Recipients: Report From the 2023 GTI (Infection and Transplantation Group) Annual Meeting

**DOI:** 10.3389/ti.2023.11859

**Published:** 2023-11-09

**Authors:** Alexandra Serris, Julien Coussement, Benoît Pilmis, Victoire De Lastours, Aurélien Dinh, François Parquin, Eric Epailly, Florence Ader, Olivier Lortholary, Emmanuel Morelon, Nassim Kamar, Edouard Forcade, David Lebeaux, Jérôme Dumortier, Filomena Conti, Agnes Lefort, Anne Scemla, Hannah Kaminski

**Affiliations:** ^1^ Department of Infectious Diseases, Necker-Enfants Malades University Hospital, Paris, France; ^2^ Sir Peter MacCallum Department of Oncology, University of Melbourne, Melbourne, VIC, Australia; ^3^ Equipe Mobile de Microbiologie Clinique, Groupe Hospitalier Paris Saint-Joseph, Paris, France; ^4^ Institut Micalis UMR 1319, Université Paris-Saclay, Institut National de Recherche Pour l’agriculture, l’alimentation et l’environnement, AgroParisTech, Jouy-en-Josas, France; ^5^ Assistance Publique-Hôpitaux de Paris, Service de Médecine Interne, Hôpital Universitaire Beaujon, Clichy, France; ^6^ Infectious Disease Department, Raymond-Poincaré University Hospital, Assistance Publique - Hôpitaux de Paris, Paris Saclay University, Garches, France; ^7^ Service de Chirurgie Thoracique et Transplantation Pulmonaire, Hôpital Foch, Suresnes, France; ^8^ Department of Cardiology and Cardiovascular Surgery, Hôpitaux Universitaires de Strasbourg, Strasbourg, France; ^9^ Infectious Diseases Department, Croix Rousse Hospital, Hospices Civils de Lyon, Lyon, France; ^10^ Institut Pasteur, Université Paris Cité, National Reference Center for Invasive Mycoses and Antifungals, Translational Mycology Research Group, Mycology Department, Paris, France; ^11^ Department of Transplantation, Edouard Herriot University Hospital, Hospices Civils de Lyon, University Lyon, University of Lyon I, Lyon, France; ^12^ Nephrology and Organ Transplantation Unit, Centre Hospitalo Universitraire Rangueil, INSERM U1043, Structure Fédérative de Recherche Bio-Médicale de Toulouse, Paul Sabatier University, Toulouse, France; ^13^ Service d'Hématologie Clinique et Thérapie Cellulaire, Centre Hospitalier Universitaire de Bordeaux, Hôpital Haut Lévêque, Bordeaux, France; ^14^ Service de Microbiologie, Unité Mobile d'Infectiologie, Assistance Publique - Hôpitaux de Paris, Hôpital Européen Georges Pompidou, Paris, France; ^15^ Hospices Civils de Lyon, Hôpital Edouard Herriot, Fédération des Spécialités Digestives, et Université Claude Bernard Lyon 1, Lyon, France; ^16^ Assistance Publique-Hôpitaux de Paris (Assistance Publique - Hôpitaux de Paris), Pitié-Salpêtrière Hospital, Department of Medical Liver Transplantation, Paris, France; ^17^ IAME, Infection Antimicrobials Modelling Evolution, UMR1137, Université Paris-Cité, Paris, France; ^18^ Department of Internal Medicine, Beaujon University Hospital, Assistance Publique - Hôpitaux de Paris, Paris, France; ^19^ Department of Nephrology and Kidney Transplantation, Necker-Enfants Malades Hospital, Assistance Publique-Hôpitaux de Paris, Paris, France; ^20^ Department of Nephrology, Transplantation, Dialysis and Apheresis, Bordeaux University Hospital, Bordeaux, France

**Keywords:** muli-drug resistant bacteria, antimicrobial resistance, antimicrobial stewardship, antifungal therapy, urinary tract infection

## Introduction

This year’s GTI (“Groupe Transplantation and Infection”) annual meeting was held in Paris, France in February 2023. This meeting focused on new approaches to manage infectious complications in solid organ and stem cell transplant recipients.

In this meeting report, we summarize the presentations and discussions from this annual meeting. Covered topics included new anti-infective agents and non-antibiotic approaches to manage infections due to multidrug-resistant Gram-negative bacteria, staphylococci, and fungal infections, as well as new approaches to manage symptomatic urinary tract infections and asymptomatic bacteriuria in kidney transplant recipients. Innovative approaches are needed to manage infectious complications in transplant recipients, who are at high risk of difficult-to-treat infections and side effects associated with the use of anti-infective agents.

## Management of Post-Transplant Bacterial Infections

### Multidrug Resistant Enterobacterales Infections in Solid Organ Transplantation: Current Situation and New Non-Antibiotic Approaches

Solid-organ transplantation (SOT) is the treatment of choice for patients diagnosed with end-stage organ disease, and the median survival of both recipients and grafts has significantly increased in the last years [1]. While the incidence of infections (including opportunistic ones such as cytomegalovirus [CMV]) is decreasing due to better prevention, the burden of “classical” infections linked to multidrug-resistant (MDR) bacteria especially related to Gram-negative bacilli (GNB) is increasing [2, 3]. Multidrug resistant Enterobacterales are involved in one-third of bacterial infections in SOT recipients [4]. Prior intestinal colonization with ESBL (extended spectrum beta-lactamase)-producing Enterobacterales is an essential prerequisite for the onset of infection among SOT recipients [5]. Furthermore, among patients with intestinal colonisation with MDR (multidrug resistance) Enterobacterales, prior exposure to anti-infectives appears to be a major risk factor for subsequent infection due to the colonizing strain [5]. This can be explained by an increase in intestinal density of resistant Gram-negative bacilli (commonly referred as relative fecal abundance) during antibiotic administration [6]. Antimicrobial stewardship (AMS) programs are designed to improve the quality of prescribing practices in terms of choice of antibiotic, dosage, duration, route of administration and de-escalation. Benoit Pilmis presented innovative AMS strategies aimed at limiting antibiotic-induced dysbiosis, decolonizing patients colonized by MDR Enterobacterales, and restoring a healthy microbiota [7]. The efficacy of oral colistin-neomycin in preventing multidrug-resistant Enterobacterales (MDR-E) infections in solid organ transplant (SOT) recipients have been evaluated previously in a multicentre, randomized, controlled, open-label, parallel-group clinical trial [8] but showed negative results in term of efficacy and tolerance (particularly for colistin).

Among these strategies, the exact benefits of fecal microbiota transplantation (FMT) remain unclear [9]. A multicenter randomized controlled trial (FeCeS study) evaluating the efficacy of FMT in decolonizing carriers of ESBL- or carbapenemase-producing Enterobacterales will provide an answer (NCT05035342). This indication of FMT in decolonizing patients has been evaluated in allo-hematopoietic stem cell transplant (allo-HSCT) recipients a systematic review has been recently published [10]. FMT was performed before or after HSCT but each time on a low number of patients. Decolonization was obtained in 40%–60% of cases. The majority of the included studies report FMT as a generally well tolerated procedure, with no serious adverse events. Interestingly, in the case series of Shouval et al. two patients developed bacteremia after the infusion, but targeted metagenomic sequencing demonstrated that the bacterial strains did not originate from the FMT inoculum [11].

Altogether, FMT seems an interesting option for decolonization, but the safety profile and efficacy of the procedure must be determined more strongly to better assess the role of FMT in allo-HSCT recipients.

One-promising way to protect the gut microbiota is to develop molecules to chelate or degrade the non-absorbed part of orally administered antibiotics and the fraction of oral and parenteral antibiotics excreted in the bile that reach the colon, induce dysbiosis and a decrease in richness and diversity of the microbiota. For example, ribaxamase (an orally administered beta-lactamase hydrolyzing β-lactams in the colon appears promising in Phase 2 studies although limited to β-lactam antibiotics) and DAV-132 which is a millimetric beads consisting of a core of a specific activated charcoal surrounded by a polymer coating that is insoluble during transit. The charcoal is activated in the ileum and adsorbs and thereby inactivates antibiotics in the caecum/colon [12–16]. For now, no investigation of this strategy exist in transplant recipients but its evaluation and implementation are of interest in the TOS patients, a population highly exposed to antibiotics.

### Multidrug Resistant Enterobacterales Infections in Solid Organ Transplantation: New Antibiotics

Antibiotic-resistant Gram-negative bacterial infections are the leading cause of death attributable to antibiotic resistance in Europe and worldwide. This is linked to the epidemic success of 3rd generation cephalosporins (3GC)- resistant Enterobacteriaceae. The widespread use of carbapenems to treat 3GC-resistant strains has led to the emergence of carbapenem-resistant isolates, in particular those secreting carbapenemases, with very limited therapeutic options. New molecules have recently been developed to combat carbapenem-resistant bacteria. Victoire de Lastours summarized the updated antimicrobial management of carbapenem-resistant bacteria related infection.

These include ceftazidime-avibactam, a combination of a 3GC with a new betalactamase inhibitor, avibactam. This combination is effective on strains carrying OXA 48 or KPC, but not metallobetalactamases. This molecule was granted authorization in Europe and the USA following 3 phase 3 trials in complicated intra-abdominal infections versus meropenem, as well as two trials in complicated urinary tract infections yielding non-inferiority. In a retrospective cohort study of 210 SOT recipients with carbapenemase-producing *Klebsiella pneumoniae* blood stream infections, ceftazidime-avibactam significantly increased the probability of 14 and 30 days clinical success, as compared to the best available therapy [17].

A second compound, meropenem-varbobactam, is also active against class A betalactamases (KPC) and cephalosporinases, but inactive against metallobetalactamases and oxacillinases, which limits its interest in some European coutries such as France, where KPCs are rare. Non-inferiority has been demonstrated in several trials against optimized treatment. A third molecule, imipenem-relebactam, is also active against KPCs but not against oxacillinases or metallobetalactamases. Imipenem-relebactam is also effective against carbapenem-resistant strains of *Pseudomonas aeruginosa*, but not against carbapenem-resistant *Acinetobacter baumanii* (CRAB). The molecule has been approved in France only as a last resort for the treatment of patients with no other possible therapeutic alternative, and in particular if KPC-type carbapenemase are produced.

Altogether, several choices are now available to treat KPC and OXA-48 oxacillinases which are approved in France and Europe. For carbapenem-resistant *P. aeruginosa*, ceftolozane-tazobactam is generally effective. Tolerance is generally good (as with beta-lactams), and these molecules are bactericidal. However, these molecules are not effective against metallobetalactamases nor against most CRAB, which poses major therapeutic problems. Its use was reported in a multicenter cohort study of 69 immunocompromised patients including 47 SOT, with multi-drug resistant *P. aeruginosa* infections, mostly respiratory and wound. Clinical cure was achieved in 68% and mortality was 19% [18].

A recently approved molecule, cefiderocol, is a siderophore cephalosporin which uses the bacterial iron entry machinery to achieve high concentrations inside the bacteria. It is unaffected by betalactamases, even metallobetalactamases, and acts as a Trojan horse. In pivotal trials, cefiderocol showed non-inferiority to high-dose meropenem in the treatment of gram-negative nosocomial pneumonia, except for *A. baumanii* infections, a result that remains unexplained. Cefiderocol has been marketed in Europe and the USA only as a last resort for infections caused by multi-resistant gram-negative bacteria, notably in cases of KPC and metallo-betalactamases.

This molecule therefore represents an important therapeutic hope, although it appears to have a relatively significant inoculum effect, which needs to be better studied. Finally, some cefiderocol-resistant strains have been described, combining several resistance mechanisms. To date, very few data are available in specific immunocompromised settings including solid organ transplantation [19], hematological malignancies [20, 21]. Most Cefiderocol prescriptions have primarily targeted multi-resistant severe *P. aeruginosa* infections, but its use has broadened to other difficult-to-treat non-fermentative gram negative bacteria, especially *S. maltophilia* for which its complex virulence and resistance profile drastically limit available antibiotics. Updated clinical and safety outcome data are needed in highly susceptible immunocompromised settings.

Another interesting combination in this context is ceftazidime-avibactam + aztreonam for strains carrying metallo-betalactamases. Several studies have demonstrated the efficacy of the avibactam + aztreonam combination, which is currently being developed by the manufacturer. An inoculum effect could also have an impact on the efficacy of this combination. This combination proved effective and safe in a serie of 4 SOT recipients with metallo-β-lactamase carbapenemase-producing Enterobacteriaceae [22].

Lastly, plazomicin, an aminoglycoside developed for the treatment of carbapenem-resistant Enterobacteriaceae infections, had shown interesting results in the United States, but was not developed in Europe due to its low commercial potential.

Treatment recommendations for carbapenem-resistant infections are summarized in the 2022 ESCMID guidelines [23]. Several new molecules are under development and could be of interest for the treatment of these infections, particularly those due to organisms producing a metallobetalactamase, such as cefepime-taniborbactam and meropenem-nacubactam. Studies are currently underway.

Finally, in the face of this type of infection, optimizing the use of available molecules is a crucial point, including rapid diagnosis of resistance, determination of MICs (minimal inhibitory concentration) for the different molecules and combinations available, and optimization of dosages with the use of high doses and prolonged infusions. Last but not least, multidisciplinary discussions between microbiologists and clinicians and the reduction of bacterial inoculum through drainage are essential. A summary of antibiotics efficiency regarding resistance mutation has been made in Table 1.

**TABLE 1:** Spectrum of new antibiotics regarding the type of resistance.

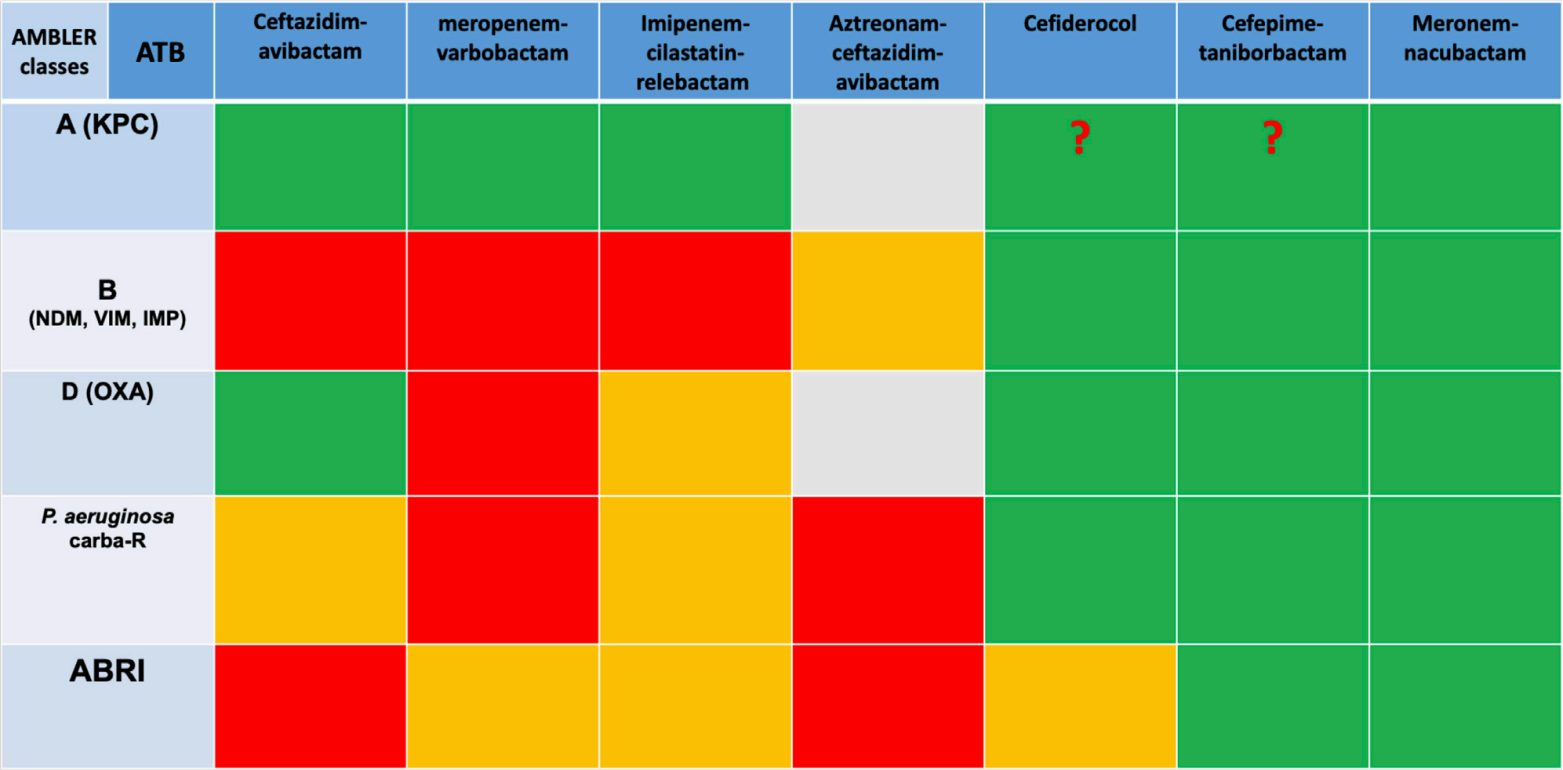

Abbreviations: ABRI, *Acinetobacter* baumani mutli resistant; ATB, antibiotic; carba-R, carbapenem-resistant.

### New Approaches to Manage Urinary Tract Infections in Kidney Transplant Recipients

The management of urinary tract infections (UTIs) in kidney transplant recipients represents a major opportunity for antimicrobial stewardship because kidney transplantation is the most common type of organ transplant worldwide, and because UTI is the most common infection in this population [3, 24]. Julien Coussement summarized the most recent evidence about the management of post-transplant symptomatic UTI and asymptomatic bacteriuria, and identified gaps of knowledge and clinical scenarios that remain understudied.

Asymptomatic bacteriuria, which is generally defined as significant bacteriuria (≥100.000 CFU/mL) without signs or symptoms of UTI (e.g., fever, chills, kidney pain, or symptoms of bladder inflammation), is relatively common after kidney transplantation [24].

Recent randomized trials have shown that the historical practice of screening for and treating asymptomatic bacteriuria is not beneficial in stable kidney transplant recipients [25–28]. A limited-size trial even suggested that asymptomatic bacteriuria might be left untreated in patients who are in the first 2 months post-transplant and have a ureteral stent [29]. Additional opportunities probably exist to improve the care of kidney transplant recipients with pyelonephritis. First, research is needed to determine the benefits and harms associated with the empiric use of very broad-spectrum antibiotics in kidney transplant recipients admitted for presumed pyelonephritis [24]. Second, a randomized trial is starting to determine whether 7 days of antibiotic therapy can be sufficient to treat non-severe episodes of pyelonephritis in kidney transplant recipients who are beyond the first month post-transplant and do not have a urinary catheter [30–32].

Besides, innovative non-antibiotic-based approaches are needed to better prevent symptomatic UTIs, which remain prevalent and detrimental after kidney transplantation. Julien Coussement discussed the potential benefits, harms and applicability of emerging approaches, including anti-adhesion therapies (which aim at preventing bacterial adhesion to host tissues, and therefore decreasing the risk of UTI) [33], intravesical instillation of a low-virulence organism (which aims at promoting bacterial interference) [34], and FMT (which aims at repopulating the gut with a “healthy” microbiome that could outcompete uropathogens) [35–38]. Vaccine candidates that are in development against extra-intestinal pathogenic *Escherichia coli* are also promising [39]. Many challenges, however, exist, including the fact that transplant recipients generally have an impaired immune response to vaccines, and the fact that around half of the UTI episodes which occur after kidney transplantation are due to microorganisms other than *E. coli.*

### New Antibiotics to Treat Infections Due to Gram-Positive Cocci

Aurélien Dinh reminded the drawbacks of vancomycin and daptomycin, before presenting new antibiotics targeting gram-positive cocci.

Vancomycin is a relatively old and difficult-to-manage glycopeptide. Several new antibiotics with activity against methicillin-resistant Staphyloc*occi* are now available.

Daptomycin is bactericidal and as effective as penicillin M against methicillin-susceptible *Staphylococcus aureus* and vancomycin for methicillin-resistant *S. aureus,* according to a randomized controlled trial (RCT) on bloodstream infections (BSI) [40]. Nevertheless, some treatment failures due to inoculum effect have been observed, and bacterial resistance is described, even among patients without previous exposure to this drug, which could be due to *in vivo* exposure to endogenous cationic peptides [41]. In liver transplant recipients, such resistance was indeed associated with prior daptomycin use and increased mortality [42]. In kidney transplant recipients, combinations of daptomycin and other antibiotics have also been suggested for resistant enterococcal infections [43, 44].

Dalbavancin is a new long acting glycolipopeptide, with a half-life of 14 days. MIC of dalbavancin against *S. aureus* and resistant coagulase-negative staphylococci are low. One retrospective cohort compared dalbavancin *versus* standard of care in patients with *S. aureus* bacteremia and found no significant difference [45]. Two RCTs are currently underway to better determine the effectiveness of dalbavancin in patients with *S. aureus* bacteremia [46, 47]. Dalbavancin is of particular interest for patients requiring prolonged antibiotic therapy, such as those with endocarditis or bone and joint infection (BJI) such as prosthetic joint infections. Several cohorts and literature reviews found dalbavancin to be safe, with nearly 80% cure rate in these indications and high level of patient satisfaction, mostly due to early discharge [48].

Ceftaroline and ceftobiprole are new generation cephalosporins with excellent activity against methicillin-resistant staphylococci according to bacterial killing curves [49]. Clinical efficacy during BJI and endocarditis are promising according to cohort studies [50, 51]. The ERADICATE trial comparing ceftobiprole *versus* daptomycin in *S. aureus* bacteremia showed non-inferiority [52].

So far, to our knowledge, no data exist regarding the use of dalvabancin, ceftaroline and ceftobiprole in SOT recipients.

Finally, oritavancin is a recently available lipopeptide, with a semi long-life activity (7 days) and important intra-cellular activity, which could be of interest for device-associated infection with biofilm [53].

These new antibiotics may allow new management and innovative approaches to treat patients with infections due to resistant Staphylococci.

## Management of Fungal Infections

Because of the toxicities of the available drugs and the emergence of resistance caused by an increased use of antifungal agents in the growing population at risk of invasive fungal diseases and in agriculture, there is a pressing need for more antifungal drug options. Recently, several new antifungal drugs have reached late-stage clinical development and obtained a temporary use authorization, as depicted by Alexandra Serris.

Olorofim is the only member of a novel class named orotomide. It inhibits fungal growth through inhibition of the fungal dihydroorotate dehydrogenase enzyme involved in pyrimidine synthesis. It has a good tissue distribution, notably in the kidney, liver, lung, and the brain (although at lower levels) [54]. It is metabolized by several CYP450 enzymes including CYP3A4 and is thus susceptible to strong CYP3A4 inhibitors and inducers. Olorofim exhibits activity *in vitro* against azole-resistant *Aspergillus, Scedosporium*, *Lomentospora*, *Rasamsonia*, dimorphic fungi (notably *Histoplasma*), dermatophytes, but has no activity against yeasts, *Mucorales* and *Alternaria alternata* [55, 56].

Olorofim is currently evaluated in two clinical studies: one open-label, single-arm study including patients with invasive fungal infections due to Lomentospora prolificans, Scedosporium spp., Aspergillus spp., and other resistant fungi with limited treatment options (ClinicalTrials.gov identifier: NCT03583164) and one phase III, randomized study to evaluate the efficacy and safety of olorofim versus liposomal amphotericin B in patients with invasive aspergillosis (ClinicalTrials.gov Identifier: NCT05101187). Published experience is currently limited to case reports (abstracts).

Ibrexafugerp is a first-in-class oral glucan synthase inhibitor, whose mechanism of action is close to the one of echinocandins (but with a different binding site). It is fungicidal against most wild-type, echinocandin or azole-resistant *Candida* spp., including *C. auris*, and fungistatic against *Aspergillus* spp [57]. Based on animal models, ibrexafungerp shows a high tissue penetration in the spleen, liver, lungs, kidney, vaginal tissue, and muscles, but not in the brain [58].

An interim analysis of the phase III FURI study evaluating the efficacy and safety of ibrexafungerp in patients with severe mucocutaneous candidiasis, invasive candidiasis, chronic or invasive aspergillosis reported complete or partial response in 58% of the patients [59]. Inclusion criteria were further expanded to include histoplasmosis, coccidioidomycosis and blastomycosis.

Rezafungin is the first member of second-generation echinocandins with enhanced pharmacokinetic/pharmacodynamic parameters, allowing for a weekly administration and potential less hepatic toxicity [60]. It has potent *in vitro* activity against most *Candida* spp., including *C. auris*, and common dermatophytes [58].

Moreover, rezafungin has shown promising results as prophylactic and curative treatment of pneumocystis *in vivo* by eradicating both the cyst and trophic forms of the fungus [61, 62]. A case report of the successful eradication of a refractory intra-abdominal candidiasis with rezafungin in a liver transplant recipient was published in 2022 [63] and rezafungin was recently found non-inferior to caspofungine in a Phase 3 trial (ReSTORE) for the treatment of candidemia/invasive candidiasis [64].

These antifungal treatments offer significant improvement in terms of spectrum of activity, tolerability, drug interactions and/or route of administration. Further clinical studies will be needed to evaluate their optimal place in the therapeutic arsenal in the solid organ transplant recipient population, taking into account the emergence of drug-resistant fungi and the problem of drug-drug interactions with immunosuppressants. Table 2 summarize the Spectrum of activity, tissue diffusion and drug-drug interactions (DDIs) with immunosuppressive drugs of olorofim, ibrexafungerp and rezafungin.

**TABLE 2:** Spectrum of activity, tissue diffusion and drug-drug interactions (DDIs) with immunosuppressive drugs of olorofim, ibrexafungerp and rezafungin.

Molecule	Spectrum of activity	Diffusion	DDIs with immunosuppressive drugs	Potential advantages
Olorofim	*Aspergillus* spp. *Scedosporium* spp. *Lomentospora prolificans Fusarium* spp. *Histoplasma capsulatum Blastomyces dermatitidis Coccidioides* spp.	• Good diffusion in kidney, liver, and lung	• Substrate of several CYP450 enzymes: anticipate dose reduction if given with a strong 3A4 inhibitor (or a moderate dual 3A4+2C9 inhibitor)	Active against highly resistant molds
		• Low levels in CNS [54]	• Weak inhibitor of CYP3A4: small reductions of tacrolimus and sirolimus might be needed (guided by standard monitoring)	
ibrexafungerp	*Candida* spp. including echinocandin resistant *C. glabrata* and *C. auris Aspergillus* spp. *Paecilomyces variotii Pneumocystis jirovecii*	• Good diffusion in liver, spleen, lungs, bone marrow, kidney, skin and uvea	• Substrate of CYP3A and P-glycoprotein: avoid coadministration of strong CYP3A inducers	• Active against resistant *Candida* species
		• Low levels in CNS [65]	• Reversible inhibitor of CYP2C8 and CYP3A4	• First orally bioavailable inhibitor of [1(3)- β-D-glucan synthase]
			• interaction with tacrolimus: 1.4-fold increase in AUC; no change in tacrolimus Cmax [66]	
Rezafungin	*Candida* spp. *Aspergillus* spp. *Pneumocystis jirovecii*	Improved drug penetration in liver and kidney abscesses (mouse model of intra-abdominal candidiasis) in comparison with micafungin [67]	Minimal inhibition of CYP450 enzymes [68]: Limited reduction (10%–19%) of the AUC or Cmax of tacrolimus, ciclosporine and mycophenolic acid (probably not clinically meaningful) [69]	• Long half-life allows once weekly dosing
				• Less hepatotoxicity
				• May prevent Pneumocystis pneumonia [61, 62]

## Conclusion

During the well-attended “**Infection and Transplantation Group**” day, the major advances in the field of **new anti-infective therapies in transplantation** were presented and discussed. New direct and indirect anti-infective approaches in transplantation are devoted to several improvements:

- decrease antibiotics pressure in our high risk multidrug resistant bacteria population with a better use of already known antibiotics and new original non-antibiotic approaches that have promising usages.
- improve efficacy of bacterial and fungal treatment with antibiotics or antifungal therapy that have a good inoculum effect and a good broadcast
- improve the tolerance of antimicrobial drugs in our polymedicated population with high risk of drugs interactions.

Altogether, those new approaches are likely to feature alternative anti-infective therapies that promise to change patient management.
